# Regulatory NK cells in autoimmune disease

**DOI:** 10.22038/IJBMS.2023.68653.14969

**Published:** 2023

**Authors:** Davood Bahadorian, Samaneh Mollazadeh, Hosein Mirazi, Tola Abdulsattar Faraj, Ramiar Kamal Kheder, Seyed-Alireza Esmaeili

**Affiliations:** 1 Immunology Research Center, Mashhad University of Medical Sciences, Mashhad, Iran; 2 Natural Products and Medicinal Plants Research Center, North Khorasan University of Medical Sciences, Bojnurd, Iran; 3 Department of Biomedical Engineering, Faculty of New Sciences and Technology, University of Tehran, Tehran, Iran; 4 Department of Basic Sciences, College of Medicine, Hawler Medical University, Erbil, Iraq; 5 Department of Medical Analysis, Faculty of Applied Science, Tishk International University, Erbil, Iraq; 6 Medical Laboratory Science Department, College of Science, University of Raparin, Rania, Sulaymaniyah, Iraq; 7 Immunology Department, Faculty of Medicine, Mashhad University of Medical Sciences, Mashhad, Iran

**Keywords:** Autoimmune disease, CD56bright NK cell, Cytokine, Immunology, Regulatory NK cell

## Abstract

NK cells are defined as the major components of the immunological network which exerts defense against tumors and viral infections as well as regulation of innate and adaptive immunity, shaped through interaction with other cells like T cells. According to the surface markers, NK cells can be divided into CD56dim NK and CD56bright NK subsets. CD56bright NK cells usually are known as regulatory NK cells. Once the immune system loses its self-tolerance, autoimmune diseases develop. NK cells and their subsets can be altered during autoimmune diseases, indicative of their prominent regulatory roles and even pathological and protective functions in autoimmune disorders. In this regard, activation of CD56bright NK cells can suppress activated autologous CD4+ T cells and subsequently prevent the initiation of autoimmunity. In this review article, we summarize the roles of regulatory NK cells in autoimmune disease occurrence which needs more research to uncover their exact related mechanism. It seems that targeting NK cells can be a promising therapeutic platform against autoimmune diseases.

## Introduction

The immune system has a pivotal role in protecting the body against invaders mediated through wonderful collaboration between diverse molecules and cells with specialized functions (1, 2). In addition to recognition of tumor cells (3, 4) and foreign molecules (5), the immune system can appropriately detect normal auto-antigens from abnormal ones and respond to the pathogens through self-tolerance mechanisms (6-8). However, the immune system may occasionally respond to auto-antigens (self-antigens) resulting in auto-reactivity followed by autoimmune disease (9). Although auto-reactivity occurs in every subject, its development can be prevented by immune regulatory cells in 90–95% of cases (10-12). Immune regulatory cells consist of type-1 regulatory T cells (T_regs_), regulatory B cells (Bregs), and innate cells such as natural killer (NK) cells (10, 13), the impaired function of which leads to production of autoantibodies against self-organs and auto-antigens (14, 15). There are at least 80 examples of autoimmune diseases, including systemic lupus erythematosus (SLE) and rheumatoid arthritis (RA) (12, 16). Epidemiologic studies are in favor of increased incidence rate of autoimmune diseases worldwide (17) with a higher risk of prevalence among women than in men (16, 18). In addition to adaptive immunity, recent evidence confirms the association of innate immune response with autoimmunity (19). Inflammatory episodes can be found nearly in all kinds of autoimmune disorders such as RA and SLE (20, 21). 

**Figure 1:**
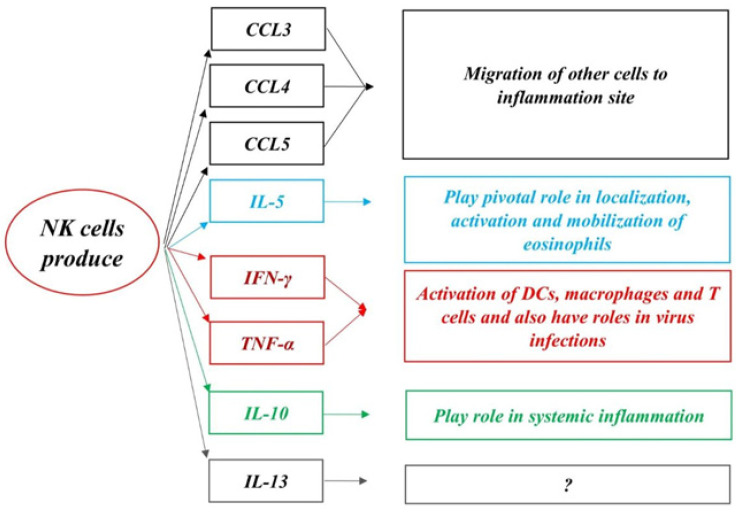
NK cells can secrete different cytokines and chemokines, subsequently affecting immune responses. NK cells by secretion of CCL3, CCL4, and CCL5 mediate migration and infiltration of other immune cells to inflammation sites. NK cells, by secretion of IL-5 affect activation, localization, and mobilization of eosinophils, and also NK cells impress activation of DCs, macrophages, and T cells by secretion of IFN-γ and TNF-α. Furthermore, NK cells affect virus infection by secretion of IFN-γ and TNF-α. NK cells play pivotal roles in systemic inflammation by production of IL-10. Although production of IL-13 by NK cells has been proven no role has been found for it by NK cells

Patients with SLE experience chronic inflammation (22, 23). It has been reported that activated NK cells can eliminate pathogenic T cells in collagen-induced arthritis (CIA) model of RA, indicative of their protective effects against disease development (24). Similarly, NK cells may have critical functions in SLE by direct killing of dendritic cells (DCs)(25, 26). NK cells are classified as the main contributors to the innate immunity network in autoimmune diseases (27), which has been discovered by Kiessling and Herberman as large granular lymphocytes in the 1970s (28). These effector lymphocytes (28-32) are best known for their protective effects against malignant cells as well as pathogens- and virus-infected cells (33). In addition, they manage immune response through interaction with other immune cells such as macrophages, DCs, T cells, and endothelial cells (30, 33, 34). NK cells can shape the adaptive immune system by interferon-gamma (31). These cells are mediators of innate and adaptive arms of the immune system (35). NK cells have inhibitory and activating receptors encoded by the germline (32, 35). Activation of NK cells is triggered by an imbalance between signals of inhibitory and activating receptors which determines the function of NK cells (36). NK cells can be considered a double-edged sword, as they can limit or exacerbate immune response (30). As shown in Figure 1, NK cells have functions in degranulation and (35)production of cytokines such as interferon-gamma (IFN-ϒ), tumor necrosis factor-alpha (TNF-α), and interleukins (IL-5, IL-10, and IL-13). Also, they aid in the migration of other effector cells to inflamed sites through secretion of chemokines, including C-C Motif Chemokine Ligand 3 (CCL3), CCL4, and CCL5 (26, 32). IL-5 secreted by NK cells contributes to localization, activation, and mobilization of eosinophils(37, 38). TNF-α and IFN-ϒ produced by NK cells play prominent roles in activation or maturation of DCs, macrophages, and T cells alongside clearance of viruses (32). IL-10 released by NK cells affects systemic inflammation(39). All NK cell-secreted factors are involved in the regulation of immune responses (32). Development of NK cells occurs first in the fetal liver and after birth in the bone marrow (31). NK cells are divided into different subsets with separate development processes according to their phenotype and functional characteristics (40). As mentioned above, NK cells have multi-faceted features and this review will especially focus on contribution of regulatory NK cells in auto-immune diseases (Figure 1).

***Human NK cell subsets***

*CD56* ^bright^ *and CD56* ^dim^ *NK cell subgroups*

Human NK cells are divided into two subsets according to the density of CD56 (known as neural adhesion molecule) on their surface (10, 40) as follows: 

*CD56* ^bright^ *NK cell subset*

This subset of NK cells has a strong expression of CD56 and weak or no expression of CD16 (CD56^bright^ CD16^dim/negative^) along with presentation of NKG2A (an inhibitory receptor) in contrast to killer cell immunoglobulin-like receptors (KIRs) (10). The number of CD56^bright^NK cells in the peripheral blood of healthy persons is about 10% (10, 40). This subset is mainly localized in the secondary lymphoid tissues because of displaying lymphocyte-expressed receptors, CCR7 and CD62L, on their surface (that promote entering the secondary lymphoid tissues) (10, 40). Inconsistently, this subpopulation does not express chemokines like CXCR1, CXCR2, and CX3CR1 mediating lymphocytes migration to the infected or inflammatory sites (40), CD56^bright^ NK cells produce cytokines such as granulocyte-macrophage colony-stimulating factor (GM-CSF), TNF-α, IFN-ϒ, IL-10, and IL-13. Similar to another study(10), there are lines of evidence showing that CD56^bright^ NK cells act as regulatory cells (40) because of their function in immune homeostasis in both normal and pathological conditions as well as their ability to regulate immune responses through cytokine secretion. Also, CD56^dim^ NK cells differentiated from CD56^bright^NK cells have few regulatory roles (10, 40). Theoretically, CD56^bright^ NK cells migrate and remain in lymph nodes (site of immune response development) and exert their regulatory functions on adaptive and innate immune cells (10) through interaction with endogenous T cells and immature DCs mediated by activator cytokines (such as IFN-ϒ) or signals (40, 41). Killing immature DCs is called DC editing (10). The proliferation of CD56^bright^ NK cells can be stimulated by activated antigen-presenting cells, (APCs)-produced IL-12, IL-15, and IL-18 having receptors on CD56^bright^ NK cells (10). (Tables 1 and 2). 

*CD56* ^dim^ *NK cell subset*

This subpopulation of NK cells slightly displays CD56 and exclusively expresses CD16 (CD56^dim^, CD16^bright^, or CD56^dim^ CD16^+^) (40). In the peripheral blood of healthy persons, the amount of CD56^dim^ NK cells is about 90% (10, 40). Moreover, CD56^dim^ NK cells are more cytotoxic and have higher expression of Ig-like NK receptors compared with CD56^bright^ NK cells. Regarding cytokine production, CD56^dim^ NK cells produce GM-CSF, TNF-α, IFN-ϒ, IL-10, and IL-13 (40). The aforementioned information brings to light that CD56^dim^ NK cells have more cytotoxicity and low regulatory effects in comparison with CD56^bright^ NK cells (Table 2). On the other hand, transcription factors Eomes and T-bet, being necessary for granule constituents’ expression, have been highly expressed on both CD56^bright^ and CD56^dim^ NK cells. Also, CD56^bright^ NK cells can better inhibit the proliferation of autologous CD4^+^ T cells by killing them (Tables 1 and 2) (10). 

*CD27* ^+^ *CD27* ^-^ *subsets*

In humans and mice, NK cells can be classified into CD27^+^ and CD27^–^ NK cells based on CD27 expression on their surface (40). Aspects of CD27^+^ CD27^- ^NK cells, according to the density of CD27 and CD11b, NK cells can be divided into four subgroups as follows; CD27^high^ CD11b^high^, CD27^low^, CD11b^low^, CD27^low^, CD11b^high^, CD27^high^, and CD11b^low^. CD27^low^ and CD11b^high ^NK subpopulations are highly cytolytic, while cytolytic effects of CD27^high^, CD11b^high^, CD27^high^, and CD11b^low^ subsets are almost equivalent to that of CD56^bright^ subset and have the capacity to secrete more cytokines and migrate to tissues (16). These data highlight the regulatory role of CD27^high^ and CD11b^high^, and CD27^high^ and CD11b^low^ NK cells (40). Taken together, the regulatory functions of CD27^high^ and CD27^low^NK cells are respectively comparable with those of CD56^bright^ and CD56^dim^NK cells (Table 2).

*Tissue-resident NK cells (trNK cells)*

For the first time, tissue-resident NK cells (trNK cells) were recognized in murine liver and then after they were found in the skin, salivary glands, kidney, uterus, and adipose tissue. Contrary to the probable regulatory role of salivary gland trNK cells in local homeostasis (42), trNK cells have limited regulatory roles overall (40) (Table 2).

***Role of regulatory NK cells in autoimmune diseases***

*Multiple sclerosis (MS)*

**Figure 2:**
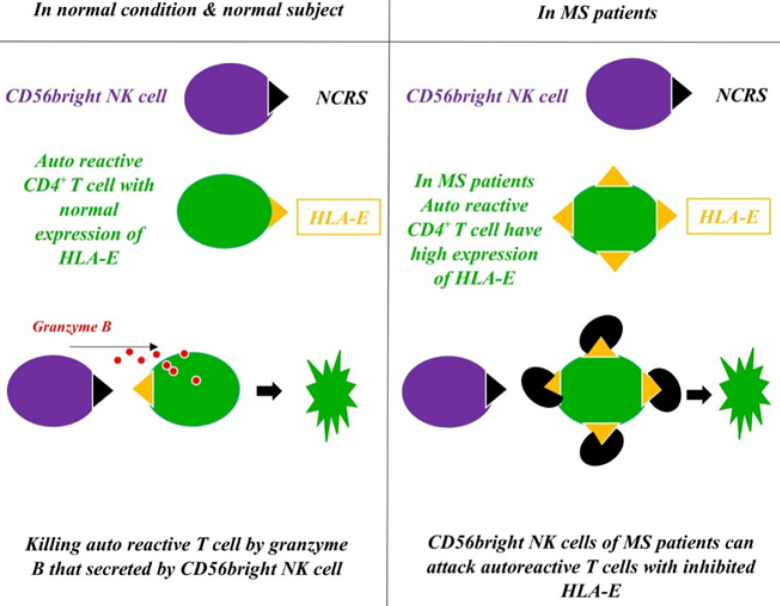
Regulatory role of CD56bright NK cells in normal conditions and an approach for the treatment of MS. In normal conditions, CD56bright NK cells by granzyme B and their NCRs eliminate autoreactive CD4+ T cells but in MS patients it seems that because of the hyper-expression of HLA-E, NK cells cannot eliminate autoreactive CD4+ T cells. Under the conditions of inhibition of HLA-E on the autoreactive CD4+ T cells, CD56bright NK cells can eliminate them

In MS, auto-reactive T cells’ response to the central nervous system (CNS) auto-antigens resulted in inflammatory damages in CNS regions evidenced by chronic inflammation and neuronal demyelination (43-46). Although the adaptive immune system has a fundamental role in MS pathogenesis, innate immunity elements like NK cells may be substantially involved in the regulation and pathogenesis of MS (46, 47). Laroni *et al*. in 2011 indicated that IL-27-treated and activated CD56^bright^ NK cells suppress the proliferation of CD4^+^ T cells compared with CD56^dim^ NK cells (48). In another similar study, Laroni *et al*. in 2016 reported that CD56^bright^ NK cells activated by IL-12 and IL-15 inhibit the propagation of autologous CD4^+^ T cells compared with CD56^dim^. Mechanistically, the suppressor activity of CD56^bright^ NK cells is mediated by enhanced gene expression of granzyme B and natural cytotoxicity receptors (NCRs). In fact, CD56^bright^ NK cells just degranulated in presence of activated CD4^+^ T cells. In the pathological condition, enhanced expression levels of major histocompatibility complex, class I, E (HLA-E) on CD4^+^ T cells CD56^bright^ NK cells diminish the inhibitory impact of autologous T cells. In contrast, CD56^bright^ NK cells of MS patients can gain their cytotoxic function by alleviation of HLA-E expression (49) (Figure 2).

***Psoriasis***

Psoriasis is classified as an autoimmune disease characterized by chronic inflammation of the skin and joints alongside rapid buildup of keratinocytes leading to scaly plaques in variable sizes (50-52). Initiation and development of psoriasis are thought to be precipitated by the pathologic contribution of innate and adaptive immune responses resulting in production of chemokines, cytokines, and growth factors. APCs like DCs have key roles in the early stages and initiation of psoriasis(51, 53). In addition to the importance of IFN-ϒ and TNF-α in psoriasis (54), IL-21 affects both innate and adaptive immune responses involving enhancement of NK cell expansion and inhibition of presenting DCs antigen. Among different NK cell subsets, CD56^bright^ NK cells proportionally contribute the most to psoriasis lesions (55) and are known as tissue-resident NK cells because of expression of the inhibitory receptor NKG2A (54-57). In contrast, the number of NK cells is decreased in the peripheral blood of psoriasis patients (55, 56, 58). CD56^bright^ NK cells play a regulatory role in the development of inflammation and chronic conditions. These cells are predominant in secondary lymphoid organs and inflamed tissues. CD56^bright^ NK cells have uniform NKG2A antigens but do not express KIRs. The reason why inflammatory cells migrate to the intra-epidermal site is linked with the expression of chemokine and adhesion molecules on stimulated keratinocytes (58). In the secondary lymphoid organs, the adaptive immune responses are shaped by not only interaction between CD56^bright^ NK cells and recently emigrated DCs, but also regulation of helper T cell polarization by CD56^bright^ NK cells mediated through releasing type-1 cytokines. In psoriatic skin, the elevated number of mature DCs leads to hyper-activation of many NK cells followed by production of high amounts of IFN-ϒ, which then affect resident cells and keratinocytes. Interaction between keratinocytes and IFN-ϒ plays an important role in amplification and chronicization of psoriasis (54). Thus, NK cells have a key role in the pathogenesis of psoriasis because of the increase in migration of NK cells especially CD56^bright^ NK cells in psoriatic skin (55). Infiltrated NK cells in the psoriatic skin secrete high levels of IFN-ϒ and little amounts of TNF-resulting in the activation of inflammatory functions of keratinocytes (Tables 3 and 4) (54).

***Systemic lupus erythematosus (SLE)***

SLE is an inflammatory auto-immune disease that may affect the skin, blood, lungs, heart, and nervous system (23, 27, 59). Similar to other auto-immune conditions, researchers believe that the NK cells have substantial roles in the pathogenesis of SLE (60). In 2009, researchers reported that the density of CD56^bright^ NK cells in the peripheral blood of active SLE patients was increased regardless of disease activity because of increased expression of type one interferon (NKp46/CD335) on CD56^bright^ NK cells more than that of CD56^dim^ subset (61). A study in 2021 reported that the content of NK cells in the peripheral blood of SLE patients was significantly less than in healthy subjects; however, the amounts of these cells were similar in inactive and active SLE patients with no difference between the number of CD56^bright^ and CD56^dim^ NK cells. The proportion of CD56^dim^ and CD56^bright^ NK cells out of total NK cells tends to be respectively reduced and enhanced in SLE patients compared with healthy persons in a non-significant manner. Analysis of activating and inhibitory receptors indicates that just CD56^dim^ NK cells are activated in active SLE patients more than in inactive subjects. Although NKp46- expressed CD56^dim ^NK cells were significantly higher in inactive SLE patients than in healthy persons; the release of NKp46by CD56^bright^NK cells was not different in either case. Production of IFN-ϒ was meaningfully increased by CD56^dim^ NK cells in SLE patients (both in active and inactive subjects) more than in healthy persons. Nevertheless, production of IFN-ϒ by CD56^bright ^NK cells had no significant differences between healthy persons and SLE patients. Compared with healthy persons, a few CD56^dim^NK cells in active SLE patients express CD158 a/h/g. Besides, decreased expression of CD158 a/h/g on CD56^dim ^NK cells in inactive patients is associated with disease activity. Despite up-regulation of CD69, the expression of activating and inhibitory markers on CD56^bright ^NK cells is not different between SLE patients and healthy persons (60). Comparably, Qing *et al*. reported that the population of NK cells in patients with SLE is lower in comparison with healthy persons(62). Obtained data have documented that the number of CD56^bright^ NK cells is enhanced in the peripheral blood of active SLE patients, while the amount of CD56^dim^ NK cells is reduced non-significantly.

Taken together, NK cells have a key role in the pathogenesis of SLE through up-regulation of cytokines and lessened cytotoxicity of NK cells in SLE patients, which make CD27^High^, CD11b^Low^, and CD56^bright^ immature NK cells the major phenotype in peripheral blood of SLE patients (63). CD56^bright^ NK cells cooperate in the development of SLE (27). High level of IL-15 in the serum of SLE patients is related to activation of DCs. Also, IL-15 manages the proliferation and contribution of NK cells in SLE through expression of Ki67. By the way, the detailed function of NK cell subsets in SLE has not been fully brought to light (27) (Tables 3 and 4).

***Rheumatoid arthritis (RA)***

**Figure 3:**
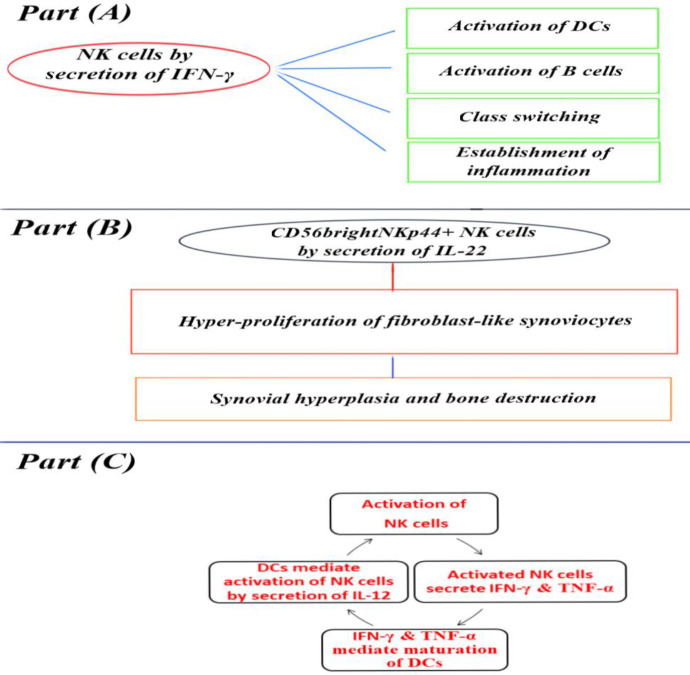
CD56 bright NK cells in RA. (A) NK cells by secretion of IFN-γ can activate DCs & B cells, class switching, and inflammation. (B) Role of CD56bright NK p44+ NK cells in the pathogenesis of RA by secretion of IL-22. (C) Interactions between NK cells and DCs

Rheumatoid arthritis (RA) is an autoimmune disorder in which the inflammatory process affects primarily synovium associated with erosion and deformity of joints. In contrast to infectious diseases, pathogenically T cells, B cells, APCs, and cytokines altogether contribute to the development of RA. Recently, researchers have found that NK cells also have noticeable roles in RA (64-66). In this context, the density of NK cells out of total lymphocytes is significantly higher in RA patients than in healthy persons(67). Regarding this fact, CD56^bright ^NK cells are present in synovial fluid as the biggest subset of NK cells (67-69). The activity of NK cells can be affected by decreased expression of activating receptors, CD244, CD16, and NKG2D, on the surface of peripheral NK cells (67). Studies indicate the inhibitory and pathogenic effects of NK cells in RA. Pathogenesis of RA mediated via NK cells is through the production of inflammatory mediators and communication with other cells in the synovium. It has been recognized that there was no difference between the ratio of CD56^bright^ to CD56^dim^ NK cells among RA patients and healthy persons (67). CD56^bright ^NK cell subset is known as a producer of cytokines such as TNF-α involved in the pathogenesis of RA. NK cells may drive inflammation in RA by production of IFN-ϒ followed by promoting activation of DCs and B cells and also class switching. Both IFN-ϒ and TNF-α released by activated NK cells can promote DC maturation. In turn, NK cells can be activated by IL-12 produced by DCs. *In vitro* studies pinpointed the protective role of NK cells in RA evidenced by the cytotoxic potential of IL-15-activated NK cells against autologous osteoclasts (67, 70, 71). Osteoclasts, derived from monocyte-macrophage lineage, are bone-resorbing cells that secrete proteinase, as well as matrix enzymes (67, 70) and regulate the homeostasis of bones (70). Treatment with tocilizumab (an inhibitor of IL-6 receptor) and rituximab increases the CD56^dim^ NK cell counts (known as cytotoxic NK cells), suggestive of the probable protective role of CD56^dim^ NK cells in RA pathogenesis (67). Besides, CD56^bright^ NKp44+ NK cells are increased in both synovial fluid and peripheral blood of RA patients and hyper-proliferate fibroblast-like synoviocytes via secretion of IL-22 leading to synovial hyperplasia and bone destruction (67) (Figure 3) (Tables 3 and 4).

***Inflammatory bowel disease (IBD)***

Two main forms of IBD are Crohn’s disease (CD) and ulcerative colitis (UC). Both chronic auto-inflammatory conditions affect the gastrointestinal tract. Over recent years, the incidence rate of IBD has increased. Since it is thought that NK cells are engaged in IBD, understanding their exact role opens a promising avenue for IBD treatment (72). A study in 2021 reported that NK cells have lower cytotoxicity function, similar levels of granzyme B, and different cytokine secretion in IBD patients compared with healthy controls. In addition, they found that CD56^bright^ NK cells produced decreased levels of IFN-ϒ and increased amounts of TNF-α and IL-17A which have pivotal impacts on IBD pathogenesis. Besides, it was revealed that the metabolic signaling of NK cells in IBD patients may be deficient and incomplete. And also CD56^bright^ NK cells of IBD patients significantly produce lower levels of IFN-ϒ than CD56^bright^ NK cells of healthy controls. They also reported that NK cells of both IBD patients and healthy controls have similar phenotypes and receptors (such as CD94, NKG2D, TRAIL, 2B4, NKp46, NKp30, and NKp44). Comparison between peripheral blood of IBD patients and healthy donors indicated that the percentage of CD56^bright^ NK cells and CD56^dim^ NK cells are similar in both IBD and healthy donors (Table 4) (72).

***Crohn’s disease (CD)***

Crohn’s disease is a chronic auto-inflammatory disorder affecting the gastrointestinal tract (72). Human gut-associated NK cells are a unique subset of mucosal NK cells that are different from conventional NK cells. Gut-associated NK cells play important roles in local immunity and express CD127 (IL7Rα), transcription factor retinoic acid-related orphan receptor C (RORC), and NKp44 or NKp46. The role of NKp44^+^ NK cells, producing IL-22 in CD, is controversial and could be either protective or pathogenic. NKp46^+^NK cells are remarkable in the intestinal mucosa of CD patients compared with healthy donors. These cells are activated by IL-23 and produce IFN-ϒ. Despite CD56^+^CD127^+^ NK cells, CD56^+^CD127^-^ NK cells induce high levels of IFN-ϒ. It was reported that polymorphism of KIRs contributes to the pathogenesis of CD (Table 3) (73).

A recent study described that the percentage and absolute numbers of peripheral blood NK cells, including CD56^dim^CD16^-^ and CD56^-^CD16^+^ NK cells, are increased in CD patients compared with healthy control subsets. In comparison, the percentages of CD56^bright^CD16^-^ and CD56^dim^CD16^+^ NK cells were decreased. Besides, absolute numbers of CD56^dim^CD16^+^ NK cells were increased in spite of CD56^bright^CD16^-^NK cells. Also, a significant increase was observed in the number of CD56^bright^CD16^+ ^NK cells rather than their percentage in the peripheral blood of CD patients. Furthermore, a subpopulation of CD56^dim^ NK cells that has a low density of CD16 (CD56^dim^CD16^dim^) was expanded in the peripheral blood of CD patients. Comparison of the expression of KIRs and other markers such as CD69, NKG2C, NKG2D, and IL-23R on peripheral blood NK cells is indicative of more activity and the important role of these cells in the development of CD. Consistently, comparison of the expression of CD107a on the surface of unstimulated NK cells manifested a high-level secretion of CD107a in CD patients compared with healthy persons, which emphasizes more degranulation of NK cells in CD patients. It was also exhibited that abnormal level of NK cell markers in CD patients shifts to normal mode after treatment (74).

**Table 1:** Comparison of the features between CD56bright and CD56dim NK cells

**Feature**	**CD56** ^bright^ ** NK cells**	**CD56** ^dim^ ** NK cells**
Density of CD56 on their surface	High (10)	Low (40)
Density of CD16 on their surface	-/Low (10)	High (40)
In peripheral blood	About 10% (40)	About 90% (40)
In secondary lymphoid tissues	About 90 % (40)	About 10% (40)
Expression of Eomes and T-bet	High (40)	High (40)
Production of IFN-ϒ	+ (10)	+ (10)
Production of TNF-α	+ (10)	+ (10)
Production of IL-10	+ (10)	+ (10)
Production of IL-13	+ (10)	+ (10)
Production of GM-CSF	+ (10)	+ (10)

**Table 2:** NK cells with regulatory functions

**Type**	**Regulatory functions**
CD56^bright^NK cells	high regulatory function (40).
CD56^dim^NK cells	low regulatory function (10).
CD27^high^ CD11b^high^NK cells	equivalent CD56^bright^NK cells ( high regulatory function) (40).
CD27^high^ CD11b^low^NK cells	equivalent CD56^bright^NK cells ( high regulatory function) (40).
CD27^low^ CD11b^high ^NK cells	equivalent CD56^dim^NK cells ( low regulatory function) (40).
Tissue-resident NK cells	limited regulatory function (40).

**Table 3:** Proportion of NK cells at the injured site in autoimmune diseases

**Disease**	**NK cells which have the highest proportion at the injured site**	**Key cytokines**
Psoriasis	CD56^bright^ in psoriatic lesions (55).	TNF-α & IFN-ϒ (54)
Rheumatoid arthritis (RA)	CD56^bright^ in synovial fluid (68).	TNF-α (75)
Crohn^’^s disease (CD)	NKp46^+^ NK cells at intestinal mucosa (73).	TNF-α & IL-17 (72)

**Table 4:** Regulatory NK cells in autoimmune diseases

**Disease**	**Role of regulatory** **NK cells in the pathogenesis of disease**
**MS**	CD56^bright ^NK cells are lower suppressors of autologous T cells that is because of increased expression of HLA-E on CD4^+^ T cells (49).
**Psoriasis**	CD56^bright ^NK cells play a regulatory role in the development of inflammation and shifting to chronic conditions in psoriatic lesions (76).
**Systemic lupus erythematosus** **(SLE)**	In SLE patients, CD56^bright ^NK cells are not active and CD56^dim^NK cells are hyperactive and produce more IFN-ϒ, while there is no difference between production levels of IFN-ϒ in SLE patients and healthy persons (60).
**Rheumatoid arthritis (RA)**	CD56^bright ^NK cells produce more TNF-α (TNF-α has a key role in the pathogenesis of RA) (67).
**Inflammatory bowel disease (IBD)**	CD56^bright ^NK cells produce a lower level of IFN-ϒ (IFN-ϒ have a key role in the pathogenesis of IBD) (72).

## Conclusion

Throughout this review, various insights have been addressed into not only pro regulatory role of NK cells in the initiation and development of autoimmune diseases, but also their protective and pathogenic roles in autoimmunity. Amongst CD56^bright^ NK cells have fundamental regulatory impacts, while CD56^dim^ NK cells and tissue-resident NK cells sometimes play regulatory roles. Although the mechanism by which NK cells exert their regulatory role in autoimmune diseases needs to be identified, studies indicate that activated CD56^bright^ NK cells kill autologous CD4^+^ T cells via cell-to-cell interaction and releasing granzyme B. In abnormal conditions, CD56^bright^ NK cells can not kill autologous CD4^+^ T cells because of the increased expression of HLA-E on CD4^+^ T cells. Future research on the role of regulatory NK cells may help to fabricate and develop desirable therapeutic approaches to manage autoimmune diseases in a more targeted fashion, such as masking HLA-E on CD4^+^ T cells to kill activated autologous T cells.

## Authors’ Contributions

DB, RKK, and HM participated in data collection and manuscript writing. SM and TAF participated as grammatical editors. SAE designed and drafted the article. All authors have fully read and approved the final manuscript.

## Conflicts of Interest

The authors declare no competing financial or non-financial interests.
